# Cardiovascular magnetic resonance evaluation of soldiers after recovery from symptomatic SARS-CoV-2 infection: a case–control study of cardiovascular post-acute sequelae of SARS-CoV-2 infection (CV PASC)

**DOI:** 10.1186/s12968-021-00798-1

**Published:** 2021-10-07

**Authors:** Daniel E. Clark, Jeffrey M. Dendy, Dan L. Li, Kimberly Crum, Debra Dixon, Kristen George-Durrett, Amar P. Parikh, Jean W. Wassenaar, Sean G. Hughes, Jonathan H. Soslow

**Affiliations:** 1 Division of Cardiovascular Medicine, Department of Internal Medicine, Vanderbilt University Medical Center, Nashville, TN USA; 2 Thomas P. Graham Division of Pediatric Cardiology, Department of Pediatrics, Monroe Carell Jr. Children’s Hospital at Vanderbilt, Nashville, TN USA; 3 Vanderbilt University Medical Center, 2220 Pierce Avenue, 383 Preston Research Building, Nashville, TN 37237 USA

**Keywords:** COVID-19, Myocarditis, Athletes, Soldiers, Return-to-service, Sports Cardiology

## Abstract

**Background:**

Myocarditis is a potential complication after severe acute respiratory syndrome coronavirus 2 (SARS-CoV-2) infection and a known cause of sudden cardiac death. Given the athletic demands of soldiers, identification of myocarditis and characterization of post-acute sequelae of SARS-CoV-2 infection with cardiovascular symptoms (CV PASC) may be critical to guide return-to-service. This study sought to evaluate the spectrum of cardiac involvement among soldiers with cardiopulmonary symptoms in the late convalescent phase of recovery from SARS-CoV-2 compared to a healthy soldier control group, and to determine the rate of progression to CV PASC.

**Methods:**

All soldiers referred for cardiovascular magnetic resonance (CMR) imaging for cardiopulmonary symptoms following COVID-19 were enrolled and matched by age, gender, and athletic phenotype 1:1 to soldiers undergoing CMR in the year prior to the first case of COVID-19 at our institution. Demographic, clinical, laboratory, and imaging parameters were compared between groups. The diagnosis of acute myocarditis was made using modified Lake Louise criteria. Wilcoxon rank sum and chi-squared tests were used for comparison of continuous and categorical variables, respectively.

**Results:**

Fifty soldier cases and 50 healthy soldier controls were included. The median time from SARS-CoV-2 detection to CMR was 71 days. The majority of cases experienced moderate symptoms (N = 43, 86%), while only 10% required hospitalization. The right ventricular (RV) ejection fraction (RVEF) was reduced in soldier cases compared to controls (51.0% vs. 53.2%, p = 0.012). Four cases were diagnosed with myocarditis (8%), 1 (2%) was diagnosed with Takotsubo cardiomyopathy, and 1 (2%) had new biventricular systolic dysfunction of unclear etiology. Isolated inferior RV septal insertion late gadolinium enhancement (LGE) was present in 8 cases and 8 controls (16% vs. 24%, p = 0.09).

Seven of the 19 (37%) cases that completed an intermediate-term follow-up survey reported CV PASC at a median of 139 days of follow-up. Two of the 7 soldiers (29%) with CV PASC had a pathological clinical diagnosis (myocarditis) on CMR.

**Conclusions:**

Cardiovascular pathology was diagnosed in 6 symptomatic soldiers (12%) after recovery from SARS-CoV-2, with myocarditis found in 4 (8%). RVEF was reduced in soldier cases compared to controls. CV PASC occurred in over one-third of soldiers surveyed, but did not occur in any soldiers with asymptomatic acute SARS-CoV-2 infection.

## Background

Myocarditis is a leading cause of sudden cardiac death (SCD) among athletes and has been identified on autopsy in up to 20% of military recruits after SCD [1]. Cardiovascular sequelae of coronavirus disease 2019 (COVID-19) include myocardial inflammation and edema [2], and an initial case series of competitive collegiate athletes found a high prevalence of myocardial late gadolinium enhancement (LGE) and myocarditis using cardiovascular magnetic resonance (CMR) [3]. More recent cohorts of universally screened collegiate athletes suggested a low prevalence of myocarditis among these minimally symptomatic athletes (1–3%) [4, 5]. The American College of Cardiology (ACC) Sports Cardiology expert panel proposed return-to-play recommendations for competitive and master athletes, with CMR reserved for those with abnormal initial screening or ongoing cardiovascular symptoms [6]. However, there are no data on the prevalence of myocardial pathology related to COVID-19 in soldiers, nor are there any expert recommendations to guide return-to-service. Soldiers represent a unique athletic population, as the physical rigor of their training combines strength and endurance activities mixed with external stressors: environmental (ambient temperatures), mental (fatigue, sleep deficits), and emotional (injuries, casualties, and separation from friends and family for prolonged time periods). We hypothesized that soldiers who recovered from severe acute respiratory syndrome coronavirus 2 (SARS-CoV-2) infection (cases) and experienced ongoing cardiopulmonary symptoms in late convalescence would have an increased prevalence of cardiovascular pathology by CMR evaluation compared to an age- and sex-matched soldier control group (controls). We additionally surveyed soldier cases for characterization of post-acute sequelae of SARS-CoV-2 infection with cardiovascular symptoms (CV PASC).

## Methods

### Study design and patient population

Soldier cases with ongoing cardiac symptoms were clinically referred for physical examination, electrocardiography (ECG), and a contrast CMR exam with parametric mapping. Healthy soldier controls were retrospectively identified from adult subjects who had clinically performed CMR exams at our institution in the 12 months (March 2019–February 2020) preceding our region’s first case of COVID-19. Soldier controls were only included if they were found to have normal cardiac function without myocardial pathology. Soldier cases and controls were included if active duty or recent military retirement in the prior year. All soldiers routinely exceed 6 hours of strenuous activity per week, with active duty soldiers performing over 14 hours weekly in activities that include endurance (marches, runs) and strength training (obstacle courses, moving heavy equipment). Failure to meet or exceed physical training standards, including a minimum score on the various physical training events and/or failure to meet height and weight standards are grounds for separation from the military. The elite (special operative) units perform much higher levels of physical exertion as a regular part of their daily training regimen.

Punctate inferior right ventricular (RV) septal insertion LGE was not considered a pathologic exclusion criterion, as this is common among healthy athletes [4, 7]. Controls were matched by age-, gender-, and athletic phenotype to cases in a 1:1 manner. Clinical demographics, laboratory, ECG, and CMR results were stored in the REDCap electronic platform [8]. COVID-19 severity was classified according to the ACC Sports Cardiology expert consensus recommendations [6]. Mild symptoms were defined as any combination of the following: anosmia, ageusia, headache, mild fatigue, mild upper respiratory tract illness, and mild gastrointestinal illness. Moderate symptoms were defined as fever, chills, myalgias, lethargy, dyspnea, and chest tightness. Severe symptoms were defined as those requiring COVID-19-related hospitalization [6].

Our laboratory’s normal values for parametric mapping were derived from a cohort of 54 healthy controls of varying age (range 7–56 years old) and gender (N = 29 male) prospectively enrolled and then segregated into the appropriate gender and age (> 18 years and < 18 years) category for comparison. Normal ranges were defined as the mean ± 2 standard deviations. A left ventricular (LV) ejection fraction (LVEF) of 50–55% was considered normal in this cohort of competitive athletes [7, 9].

### CMR protocol

A comprehensive CMR with contrast was performed on a 1.5 T CMR system (Avanto Fit, Siemens Healthineers, Erlangen, Germany). The CMR protocol consisted of cine CMR balanced stead-state free precession (bSSFP) imaging to calculate LV and RV volumes, LVEF, and LV mass. Intravenous gadolinium contrast (gadobutrol, Gadavist®, Bayer Healthcare Berlin, Germany) at a dose of 0.15 mmol/kg) was administered through a peripheral intravenous line. LGE was performed using segmented inversion recovery (optimized inversion time to null myocardium) and single shot phase sensitive inversion recovery (inversion time of 300 ms) imaging in standard long-axis planes and a short-axis stack.

Native T1 mapping, T2 mapping, and post-contrast (15 min after contrast administration) T1 mapping was performed at the base and mid-LV in the short axis stack. All parametric mapping sequences were breath-held; any image felt to be inadequate due to poor breath holds or poor motion correction was repeated at the time of the scan. T1 mapping was performed using a sequence acquired using a 5(3s)3 protocol before contrast and 4(1)3(1)2 protocol after contrast. Modified Look-Locker inversion recovery (MOLLI) sequences were motion-corrected, ECG-triggered images obtained in diastole with typical imaging parameters: non-selective inversion with a 35 degree flip angle, single shot bSSFP imaging, initial inversion time of 120 ms with 80 ms increments, field of view 340 × 272 mm^2^, matrix size 256 × 144, slice thickness 8 mm, voxel size 1.3 × 1.9 × 8.0 mm^3^, TR/TE 2.6/1.1 ms, parallel imaging factor of 2. The matrix size was decreased to 192 × 128 for heart rates > 90 (approximate voxel size 1.8 × 2.1 × 8 mm^3^). T2 mapping was performed using a breath-held, ECG-triggered, bSSFP sequence with motion correction. Typical imaging parameters were as follows: Adiabatic T2 preparation with 35 degree flip angle, field of view 340 × 272 mm^2^, matrix size 192 × 144, slice thickness 8 mm, voxel size 1.8 × 1.9 × 8.0 mm^3^, TR/TE 2.5/1.1 ms, parallel imaging factor of 2.

### CMR post-processing

CMR post-processing was performed blinded to clinical data. Ventricular volumes and function were calculated using QMass (MedisSuite 2.1, Medis, Leiden, The Netherlands). The presence or absence of LGE, as well as location using the standard 17-segment model [10], was qualitatively assessed by two cardiologists, both with over 10 years of CMR experience. If there was disagreement, a third cardiologist assessed for LGE. Areas of LGE determined not to be due to myocardial infarction were included in the analysis, as these were felt to be the most focal areas in a continuum of diffuse extracellular matrix expansion [11]. LGE quantification was performed using the full-width half-maximum method, as this has previously been shown to have a stronger correlation with adverse outcomes in myocarditis [12]. Isolated inferior RV insertional LGE was not deemed to be pathologic as this is a common finding among athletes and was therefore not included in LGE quantification [4, 7].

T1 maps were obtained prior to and after contrast administration as previously described [13]. Extracellular volume fraction (ECV) maps were created using Qmaps (Medis) from native T1 and post-contrast T1 maps as well as a hematocrit obtained at the time of the scan. Regions of Interest (ROIs) were manually drawn on T1, T2, and ECV maps within the LV mesocardium, carefully avoiding partial volume averaging with blood-pool or epicardial fat and artifact. As per our lab protocol, ROIs were placed in the septum and free wall of the basal and mid short-axis slices, as well as in areas with focal abnormalities identified after visual inspection. Acute myocarditis was diagnosed according to the modified Lake Louise criteria [14].

### Statistical analysis

Categorical variables were compared using the Pearson’s chi-squared test or Fisher exact test and reported as frequency and percentage. Continuous variables were compared using the Wilcoxon rank sum and presented as median and interquartile range (IQR). Statistical analysis was performed using STATA (version 15 m Stata Corporation, College Station, Texas, USA). All tests were 2-sided and a p-value < 0.05 was considered significant. The study was approved by the institutional review board at Vanderbilt University Medical Center which waived informed consent.

## Results

Fifty cases and 50 healthy soldier controls met inclusion criteria with a median age of 27 years and 25 years, respectively (Table 1); 98% were male. The majority of cases (98%) and controls (98%) were active duty personnel. Eighty-six percent of soldier cases experienced acute cardiovascular symptoms with COVID-19 and 94% experienced cardiovascular symptoms during the late convalescent phase; 84% had dyspnea on exertion, 71% reported chest pain, and 18% had palpitations. The most common indication for CMR among soldier controls was an abnormal screening ECG or transthoracic echocardiogram (N = 24) followed by chest pain (N = 21), palpitations (N = 6), syncope (N = 5), or another indication (N = 5).

**Table 1:** Demographics and cardiovascular magnetic resonance (CMR) data among COVID-19 + soldiers (soldier cases) compared to healthy soldier controls

	Soldier cases (N = 50)	Soldier healthy controls (N = 50)	P value
Age, median (IQR), years	26.5 (23, 31)	25 (23, 33)	0.82
Height, cm	178 (175, 183)^a^	177 (173, 185)	0.95
Weight, kg	87 (78, 99)^a^	86 (75, 98)	0.83
Body surface area, kg/m^2^	2.1 (2.0, 2.2)^b^	2.1 (1.9, 2.2)	0.80
Gender, male	49 (98%)	49 (98%)	0.75
Active duty	49 (98%)	49 (98%)	1.00
Creatinine, mg/dL	0.9 (0.9, 1.0)^c^	1.1 (1.0, 1.2)^c^	n/a
Hematocrit, %	44 (43, 46)^a^	42 (41, 44)^d^	n/a
Acute cardiovascular symptoms, N, %	42 (86%)	n/a	n/a
(*Convalescent) CV symptoms	46 (94%)	n/a	
Dyspnea on exertion	41 (84%)	3 (6%)	< 0.001
Chest pain	35 (71%)	21 (42%)	0.005
Palpitations	9 (18%)	6 (12%)	0.401
COVID-19 severity N (%)			
Mild (no CV symptoms)	2 (4%)	n/a	n/a
Moderate (CV symptoms)	42 (86%)	n/a	
Severe (hospitalized)	3 (6%)		
Severe (ICU)	2 (4%)		
CV PASC			
Survey completion, N, %	19 (39%)	n/a	n/a
Median time to survey (days)	91		
Ongoing CV symptoms	6 (32%)		
Laboratory			
Troponin I > 99%	2 (4%)^a^	0 (0%)^e^	n/a
Abnormal ECG	4 (8.5%)^a^	9 (28%)^f^	0.05
Hematocrit	44 (43, 45)^c^	42 (41, 43)^d^	0.06

*CV* cardiovascular, *ECG* electrocardiogram, *ICU* intensive care unit, *n/a* not applicable

*Convalescent CV symptoms for soldier cases, compared to any CV symptoms for controls

^a^N = 48

^b^N = 47

^c^N = 14

^d^N = 7

^e^N = 9

^f^N = 32

Initial COVID-19 severity was mild in 4%, moderate with cardiovascular symptoms in 86%, and severe with hospitalization in 10% (N = 5), 2 of whom required ICU care. None of the hospitalized soldier cases (N = 5, 10%) had an abnormal CMR. Troponin I was normal in 96% at a median 33 days post-SARS-CoV-2 detection. Two soldier cases (4%) had troponin I > 99% of the upper reference limit of normal on multiple samples, neither of whom had an abnormal CMR. Four soldier cases (8%) had an abnormal baseline ECG, none of whom had an abnormal CMR.

The median time from SARS-CoV-2 infection to CMR in cases was 71 days (IQR 47–103; range 22–197). There were no differences in baseline demographics, laboratory findings, CMR LV parameters, or CMR parametric mapping (T1, T2 or ECV) findings between cases and controls (Table 2). Soldier cases had reduced RVEF compared to controls (51.0% vs. 53.2%, p = 0.012). While soldier cases had a trend towards increased RV volumes, this did not reach statistical significance (Table 2). Soldier cases had more myocarditis-type LGE than controls (8% vs. 0%, p = 0.03; Table 2).

**Table 2:** Imaging data among COVID-19 + soldiers (soldier cases) compared to soldier controls

	Soldier cases (N = 50)	Soldier healthy controls (N = 50)	P value
LVEF, median (IQR), %	59.5 (56.9, 63.0)	57.8 (55.7, 61.3)	0.24
LVEDV, mL	178.0 (162.2, 201.4)	188.5 (165.2, 211.6)	0.12
LVEDVI, mL/m^2^	84.9 (79.8, 94.5)	91.2 (82.6, 103.0)	0.059
LVESV, mL	70.6 (61.2, 85.6)	78.8 (61.9, 89.2)	0.17
LVESVI, mL/m^2^	34.6 (30.1, 39.4)	39.2 (31.8, 44.6)	0.025
LV mass, g	134.3 (116.1, 146.1)	122.9 (110.4, 139.5)	0.06
LV mass index, g/m^2^	64.6 (58.4, 69.1)	59.6 (55.6, 65.5)	0.023
RVEF, %	51.0 (48.5, 54.0)	53.2 (50.8, 58.7)	0.012
RVEDV, mL	200.8 (178.0, 224.8)	183.2 (168.9, 237.0)	0.33
RVEDVI, mL/m^2^	98.0 (86.9, 109.5)	93.8 (80.6, 109.5)	0.35
RVESV, mL	100.5 (85.7, 116.1)	85.3 (70.7, 110.6)	0.10
RVESVI, mL/m^2^	47.8 (42.6, 55.2)	43.1 (34.6, 51.1)	0.052
T_1_, median (IQR), ms			
Mid septum	982.2 (953.6, 991.7)	982.8 (966.0, 998.6)^b^	0.22
Mid lateral	970.7 (952.9, 985.1)	964.5 (948.3, 987.0)^b^	0.42
Mid global	971.5 (953.4, 985.8)	972.5 (961.7, 987.6)^b^	0.44
T_2_ median (IQR), ms			
Mid septum	45.3 (43.8, 46.7)^c^	44.8 (43.9, 46.3)^d^	0.71
Mid lateral	45.1 (43.7, 45.9)^c^	45.4 (44.0, 46.7)^d^	0.72
Mid global	45.3 (44.3, 46.3)^c^	45.4 (44.4, 46.3)^d^	0.69
ECV (global), %			
Mid septum	23.6 (22.1, 25.2)^c^	22.7 (21.9, 23.1)^e^	0.38
Mid lateral	22.1 (20.5, 22.9)^c^	20.8 (20.7, 22.4)^e^	0.58
Mid global	23.0 (21.3, 24.1)^f^	22.3 (22.0, 24.3)^g^	0.97
LGE			
Any, N (%)	11 (22.5%)^h^	8 (23.5%)^i^	0.56
Inferior RV septal insertion	8 (16%)^c^	8 (23.5%)^i^	0.09
Subepicardial inferolateral	4 (8%)^c^	0 (0%)^i^	0.03
Diagnosis			
Myocarditis	4 (8%)	0 (0%)	
Takotsubo cardiomyopathy	1 (2%)	0 (0%)	

*ECV* extracellular volume, *LGE* late gadolinium enhancement, *LV* left ventricle, *LVEDV* left ventricular end-diastolic volume, *LVEDVI* left ventricular end-diastolic volume index, *LVEF* left ventricular ejection fraction, *LVESV* left ventricular end-systolic volume, *LVESVI* left ventricular end-systolic volume index, *RVEDV* right ventricular end-diastolic volume, *RVEDVI* right ventricular end-diastolic volume index, *RVEF* right ventricular ejection fraction, *RVESV* right ventricular end-systolic volume, *RVESVI* right ventricular end-systolic volume index, *n/a* not applicable

^a^N = 11

^b^N = 48

^c^N = 47

^d^N = 13

^e^N = 5

^f^N = 46

^g^N = 4

^h^N = 49

^i^N = 34

**Fig. 1:**
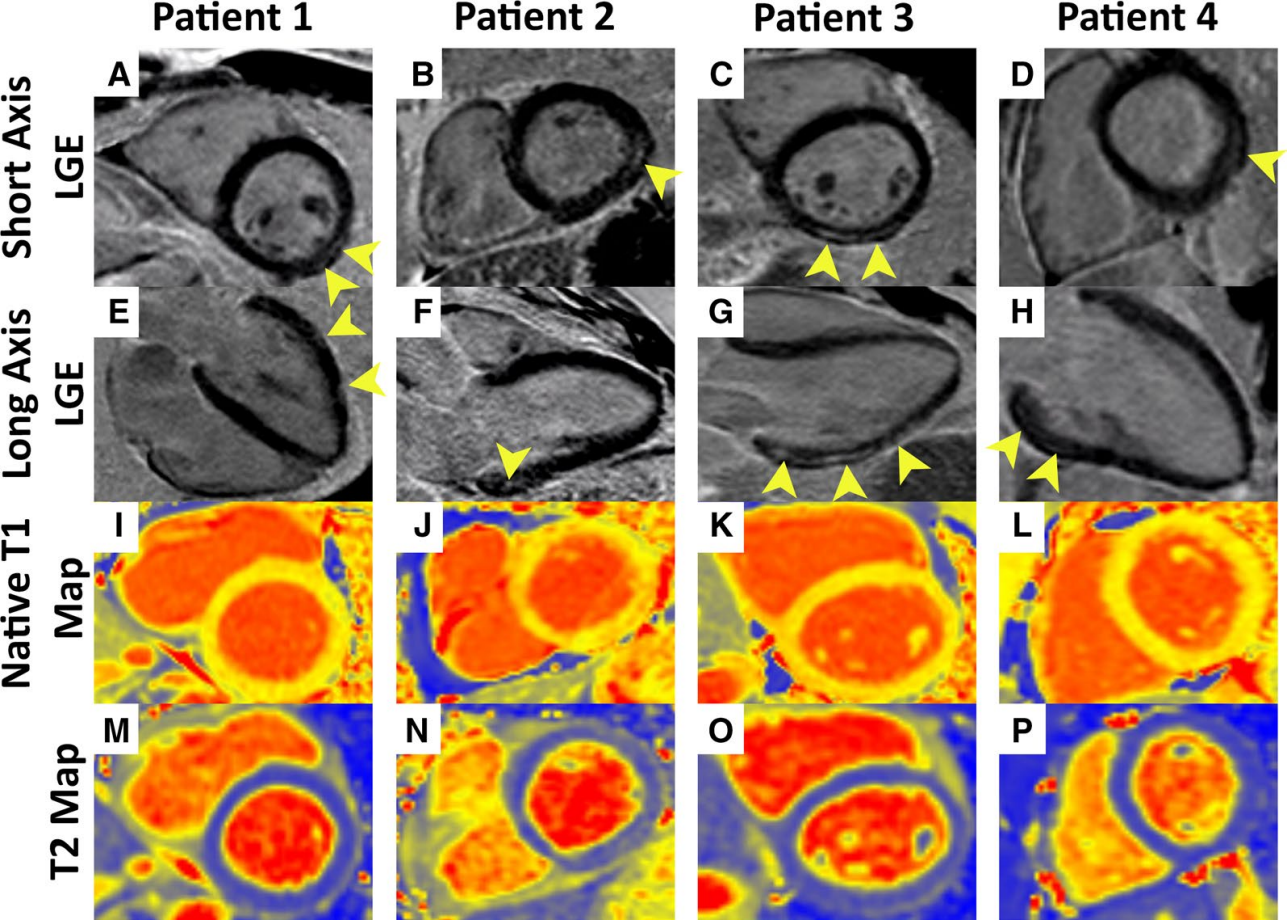
1st row (**A**–**D**): Phase sensitive inversion recovery (PSIR) images in the short-axis plane demonstrating focal areas of late gadolinium enhancement (LGE) in a subepicardial distribution typical of myocarditis (yellow arrowheads); 2nd row (**E**–**H**): long-axis PSIR images demonstrating focal areas of LGE in a pattern consistent with myocarditis (yellow arrowheads); 3rd row (**I**–**L**): native myocardial T1 map, **I**: 975 ms global base, **J**: 995 ms global base, **K**: 1010 ms global base, **L**: 980 ms global base; 4th row (**M**–**P**): myocardial T2 map, **M**: 43.0 ms global base, **N**: 45.5 ms global base, **O**: 46.5 ms global base; region of interest (basal inferolateral wall): 52.0 ms, **P**: 42.7 ms global base

**Fig. 2:**
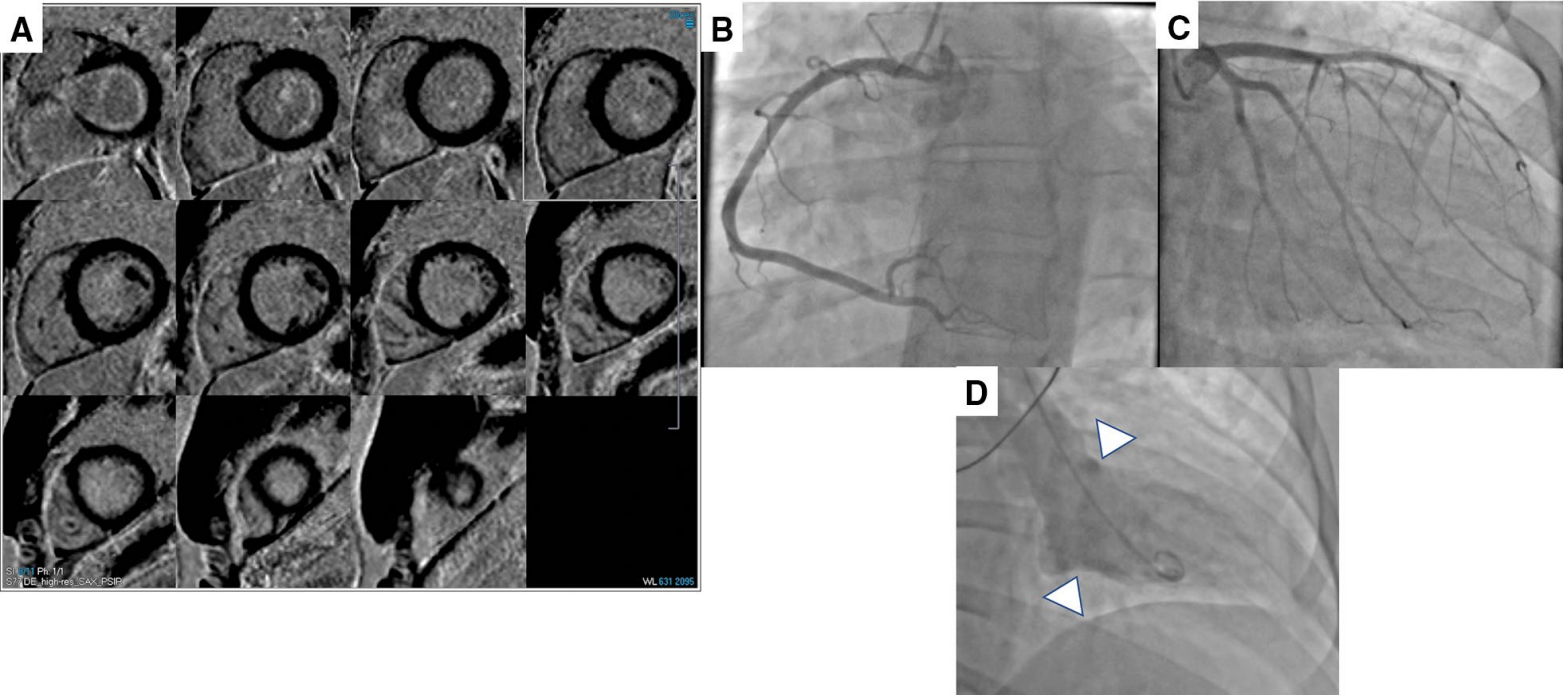
Soldier case with Takotsubo cardiomyopathy. **A** Phase sensitive inversion recovery (PSIR) short-axis images revealing no myocardial or pericardial late gadolinium enhancement (LGE), **B**, **C** Normal coronary arteries on angiography, and **D** Basal hyperkinesis with mild apical ballooning on left ventriculogram

Eleven (22%) of soldier cases had myocardial LGE. Four cases (8%) were diagnosed with myocarditis (1 acute myocarditis, 3 with healing myocarditis-pattern LGE), one of whom (2%) had concurrent pericarditis (Fig. 1). One soldier (2%) was diagnosed with Takotsubo cardiomyopathy after selective coronary angiography confirmed the absence of obstructive coronary artery disease (Fig. 2), and one soldier (2%) had new biventricular systolic dysfunction without myocarditis. All of the soldiers with myocarditis had preserved biventricular systolic function. The mean quantified pathologic LGE was 6.2% of the myocardial mass, using the full-width half-maximum method. Isolated inferior RV septal insertion LGE was present in 8 cases and 8/34 controls who received contrast (16% vs. 24%, p = 0.09).

### CV PASC

PASC with cardiovascular symptoms (CV PASC) was present in 37% of soldier cases at a median 139 days of follow-up. All soldiers with CV PASC had persistent symptoms since the initial infection; no soldiers with asymptomatic acute-COVID or with resolution of acute cardiovascular symptoms went on to develop CV PASC. Two (29%) of soldier cases with CV PASC had a pathological clinical diagnosis (myocarditis) on CMR (Table 3).

**Table 3:** Soldier cases compared by presence versus absence of post-acute sequelae of SARS-CoV-2 infection (CV PASC)

	CV PASC (N = 7)	No PASC (N = 12)	P value
LVEF, median (IQR), %	57.8 (57.1, 63.1)	57.9 (54.7, 61.5)	0.45
LVEDV, mL	168.1 (164.3, 207.7)	184.6 (168.1, 206.3)	0.61
LVEDVI, mL/m^2^	84.3 (82.5, 96.0)	84.3 (81.9, 95.2)	0.61
LVESV, mL	69.0 (64.3, 81.1)	74.6 (65.5, 92.1)	0.35
LVESVI, mL/m^2^	34.8 (32.2, 38.3)	36.4 (32.4, 40.5)	0.53
LV mass, g	136.4 (127.3, 158.2)	130.5 (126.2, 140.5)	0.47
LV mass index, g/m^2^	64.8 (62.6, 74.8)	62.5 (58.7, 66.7)	0.16
RVEF, %	50.6 (48.3, 52.0)	50.5 (46.9, 53.2)	0.87
RVEDV mL	221.0 (192.2, 276.3)	208.6 (188.3, 226.0)	0.40
RVEDVI, mL/m^2^	107.9 (94.9, 129.3)	99.6 (88.0, 105.8)	0.24
RVESV, mL	110.3 (95.1, 135.7)	105.3 (90.3, 114.4)	0.55
RVESVI, mL/m^2^	55.3 (45.6, 63.5)	48.4 (47.5, 53.5)	0.35
T_1_, median (IQR), ms			
Mid septum	986.4 (958.5, 1007.7)	985.6 (964.0, 994.1)	0.74
Mid lateral	970.2 (962.2, 983.7)	973.1 (944.6, 1001.0)	0.80
Mid global	972.1 (956.0, 1003.6)	978.5 (953.9, 996.3)	0.99
T_2_ median (IQR), ms			
Mid septum	45.7 (45.0, 47.4)	44.7 (43.8, 45.7)^b^	0.10
Mid lateral	45.5 (44.6, 45.6)	45.6 (43.8, 46.1)^b^	0.62
Mid global	45.7 (44.8, 47.5)	45.3 (44.1, 46.1)^b^	0.10
ECV (global), %			
Mid septum	25.4 (24.1, 26.1)^a^	23.4 (22.3, 25.3)^b^	0.12
Mid lateral	23.0 (22.5, 24.2)^a^	22.1 (20.5, 22.5)^b^	0.06
Mid global	23.9 (22.0, 25.7)^a^	23.0 (21.3, 23.4)^c^	0.36
LGE			
Any, N (%)	3 (42.9%)	1 (9.1%)^b^	0.25
Inferior RV septal insertion	1 (14.3%)	1 (9.1%)^b^	0.99
Subepicardial inferolateral	2 (28.6%)	0 (0%)^b^	0.14
Diagnosis			
Myocarditis	2 (28.6%)	0 (0%)^b^	0.14

^a^N = 6

^b^N = 11

^c^N = 10

There were no significant volumetric or parametric differences between soldier cases with or without CV PASC, but soldiers with CV PASC were more likely to have myocarditis-pattern LGE than those without CV PASC (Table 3).

### Follow-up CMR

All 4 soldier cases with myocarditis have undergone follow-up CMR. Case 1 had inferolateral LGE and normal T2 time on the initial CMR (33 days post-COVID-19 diagnosis) and demonstrated complete resolution of LGE (reduction of LGE burden from 8.8 to 2.8% at 82 days post-infection and complete resolution 119 days post-infection; Table 4). Case 2 had myocarditis-type LGE (3.5% burden) on the initial CMR 124 days post-infection, which remained unchanged on a follow-up CMR 271 days post-infection. Case 3 had persistent LGE (5.1% burden) and ongoing mild T2 elevation (51 ms) on repeat CMRs 122 days and 213 days following infection. Case 4 had basal-to-mid-LV inferoseptal and inferolateral myocarditis-type LGE (7.4% burden) on the initial CMR 97 days post-infection, which decreased to 2.1% on CMR 192 days post-infection, and completely resolved by CMR 245 days post-infection (Table 4).

**Table 4:** Follow-up CMR in soldier cases with COVID-19 myocarditis

Case	CMR1 (days since diagnosis)	CMR1%LGE	CMR2 (days since diagnosis)	CMR2%LGE	CMR3 (days since diagnosis)	CMR3%LGE
1	33	8.8	82	2.8	119	0
2	124	3.5	271	3.5		
3	38	5.1	122	5.1	213	5.1%
4	97	7.4	192	2.1	245	0

## Discussion

This study suggests that the prevalence of myocarditis-type LGE (8%) is considerable in soldiers with cardiovascular symptoms in the late convalescent phase of COVID-19. The focus on late convalescent CMR is critical as the pandemic transitions from management of acute infections to assessment and treatment of previously infected patients with ongoing symptoms. This study helps inform the yield of CMR among this increasingly larger group of individuals post-COVID-19 with ongoing cardiac symptoms. To our knowledge, this is the first study to report the CMR findings in soldiers after SARS-CoV-2.

Soldier cases were noted to have a reduced RVEF compared to controls, as has been reported in other publications [2, 15]. These findings are intriguing and merit consideration that COVID-19 may preferentially affect the RV either directly, or indirectly from lingering effects of COVID-19 related lung disease [16].

Athletic remodeling with increased chamber volumes and low-normal biventricular systolic function was similarly present in cases and controls. Punctate inferior RV septal insertional LGE was also common in both cases and controls. This finding is consistent with our prior report in collegiate athletes and is likely due to athletic remodeling, not myocarditis secondary to COVID-19, and again emphasizes the critical importance of an athletic control group [4, 7].

CV PASC was common among soldiers (37%) and was exclusively reported in soldiers with similar symptoms during the acute and early convalescent phases of illness. Soldiers with CV PASC trended towards having more myocarditis (28.6% vs. 0%, p = 0.14, Table 3). Other more subtle differences between these groups may not have reached statistical significance due to the small number of subjects in this sub-analysis. Follow-up CMR among myocarditis cases showed two patterns: recovery with gradual LGE resolution, and persistence with one soldier having ongoing active myocarditis with T2 elevation more than 7 months after COVID diagnosis.

## Limitations

Because CMR was performed at a median time of 71 days after infection, milder cases of myocarditis may have been missed if edema and LGE resolved quickly. The sensitivity of troponin I as a biomarker for COVID-19 myocarditis may be limited due to the necessary quarantine period and the delayed acquisition of these samples (median 33 days post-SARS-CoV-2 in soldier cases). Controls were not prospectively obtained, but rather derived retrospectively from a convenience soldier sample with clinical indications for CMR, and thus may have subtle abnormalities that would not be present in an asymptomatic, prospectively enrolled control group. However, controls were matched by age, sex, and athletic phenotype to account for known athletic cardiac remodeling and were only included if they had undergone CMR at a date prior to the first case of SARS-CoV-2 in our region, thereby eliminating concern for undiagnosed prior COVID-19. Although the survey response rate was not unreasonable (39%), smaller numbers could impact our results.

## Conclusions

Cardiovascular pathology was diagnosed in 12% of soldiers after recovery from SARS-CoV-2, with myocarditis found in 8%. Compared to matched soldier controls, RVEF is lower in soldiers after recovery from COVID-19. CV PASC occurred in over one-third of soldiers surveyed but did not occur in any soldiers with asymptomatic acute SARS-CoV-2 infection. Ongoing investigations into the cardiovascular sequelae of COVID-19 are crucial in tailoring data-driven return-to-service recommendations for our soldiers.

## Data Availability

The datasets used and analyzed during the current study are available from the corresponding author on reasonable request.

## Abbreviations

ACC: American College of Cardiology
bSSFP: Balanced steady state free precession
CMR: Cardiovascular magnetic resonance
COVID-19: Coronavirus disease-2019
CV PASC: Post-acute sequelae of SARS-CoV-2 infection with cardiovascular symptoms
ECG: Electrocardiogram
ECV: Extracellular volume fraction
IQR: Interquartile range
LGE: Late gadolinium enhancement
LV: Left ventricle/left ventricular
LVEDV: Left ventricular end-diastolic volume
LVEDVI: Left ventricular end-diastolic volume index
LVEF: Left ventricular ejection fraction
LVESV: Left ventricular end-systolic volume
LVESVI: Left ventricular end-systolic volume index
MOLLI: Modified look-locker inversion recovery (MOLLI)
PASC: Post-acute sequelae of SARS-CoV-2 infection
PSIR: Phase sensitive inversion recovery
RV: Right ventricle/right ventricular
RVEDV: Right ventricular end-diastolic volume
RVEDVI: Right ventricular end-diastolic volume index
RVEF: Right ventricular ejection fraction
RVESV: Right ventricular end-systolic volume
RVESVI: Right ventricular end-systolic volume index
SARS-CoV-2: Severe acute respiratory syndrome coronavirus 2
SCD: Sudden cardiac death
